# On learning what to learn: Heterogeneous observations of dynamics and establishing possibly causal relations among them

**DOI:** 10.1093/pnasnexus/pgae494

**Published:** 2024-12-06

**Authors:** David W Sroczynski, Felix Dietrich, Eleni D Koronaki, Ronen Talmon, Ronald R Coifman, Erik Bollt, Ioannis G Kevrekidis

**Affiliations:** Department of Chemical and Biological Engineering, Princeton University, Princeton, NJ 08544, USA; School of Computation, Information and Technology, Technical University of Munich, 80333 Munich, Germany; Faculty of Science, Technology and Medicine, University of Luxembourg, 1350 Kirchberg, Luxembourg; Viterbi Faculty of Electrical Engineering, Technion, Israel Institute of Technology, Haifa 3200003, Israel; School of Engineering & Applied Science, Yale University, New Haven, CT 06511, USA; Electrical & Computer Engineering, Clarkson University, Potsdam, NY 13699, USA; Department of Chemical and Biomolecular Engineering, Johns Hopkins University, Baltimore, MD 21218, USA; Department of Applied Mathematics and Statistics, Johns Hopkins University, Baltimore, MD 21218, USA; Department of Urology, Johns Hopkins University, Baltimore, MD 21218, USA

**Keywords:** heterogeneous observations, learning inputs, common variables, identifiability, causality

## Abstract

Before we attempt to (approximately) learn a function between two sets of observables of a physical process, we must first decide what the *inputs* and *outputs* of the desired function are going to be. Here we demonstrate two distinct, data-driven ways of first deciding “the right quantities” to relate through such a function, and then proceeding to learn it. This is accomplished by first processing simultaneous heterogeneous data streams (ensembles of time series) from observations of a physical system: records of multiple *observation processes* of the system. We determine (i) what subsets of observables are *common* between the observation processes (and therefore observable from each other, relatable through a function); and (ii) what information is *unrelated* to these common observables, therefore particular to each observation process, and not contributing to the desired function. Any data-driven technique can subsequently be used to learn the input–output relation—from k-nearest neighbors and Geometric Harmonics to Gaussian Processes and Neural Networks. Two particular “twists” of the approach are discussed. The first has to do with the *identifiability* of particular quantities of interest from the measurements. We now construct mappings from *a single* set of observations from one process to *entire level sets* of measurements of the second process, consistent with this single set. The second attempts to relate our framework to a form of causality: if one of the observation processes measures “now,” while the second observation process measures “in the future,” the function to be learned among what is common across observation processes constitutes a dynamical model for the system evolution.

**Significance Statement**

This work focuses on identifying the relevant inputs and outputs for a function in a data-driven manner. It learns this function using data from sensors that capture partial information and are affected by sensor-specific noise, without requiring knowledge of the underlying physical laws. Consequently, it is essential to extract common, relevant information from the sensors while isolating irrelevant data. Various methods are proposed to achieve this, which opens up the possibility of discovering causal relationships. This methodology is applied to the data-driven discovery of dynamical models for processes using multimodal, noisy data.

## Introduction

In recent years, the technology for observing/measuring phenomena and dynamic behavior in many disciplines, from physics and chemistry to biology and the medical sciences, has been growing at a spectacular pace—both the types of possible measurements as well as their spatiotemporal resolution and accuracy are constantly enriched. It becomes thus increasingly possible to have several different measurements of the same phenomenon, observed simultaneously through different instruments (one could, for example, measure the extent of a reaction through measuring reactant/product concentrations or through measuring a physical property—say, a refractive index—of the reacting mixture).

**Fig. 1.**
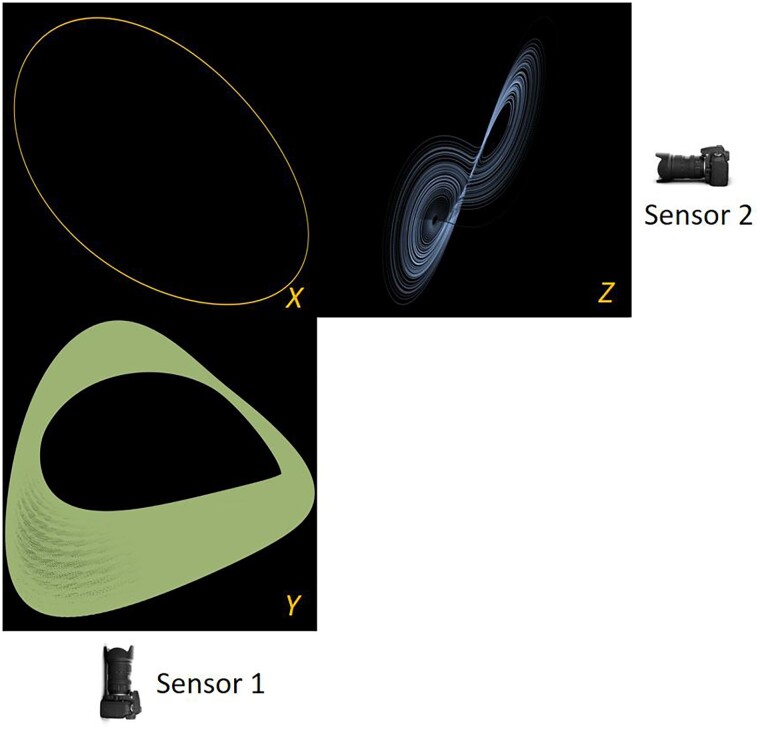
Illustrative sensor setup: sensor 1 only observes parts of systems *X* and *Y*. Sensor 2 only observes parts of systems *X* and *Z*.

The simultaneous progress in the mathematics of algorithms for data mining also open the way to registering such disparate measurements, and even fusing them. Discussions of “gauge invariant data mining” (1–6), that is, data mining that ultimately does not depend on the measuring instrument (as long as sufficiently rich information is collected) is a topic of active current research (7–10). The ability to sufficiently accurately record the covariance of measurement noise around each measurement point is known to enable powerful tools for data registration/fusion (7–15). Different measurements of the same phenomenon (by which we imply measurements by different measuring instruments/observations through different observation functions) are often contaminated by instrument-specific distortion that hinders the registration/fusion task. This distortion could be *instrument-specific noise*; alternatively (and the examples in this paper are based on this latter paradigm) each instrument may pick up, in addition to the process of interest, information from additional, unrelated processes, that take place “in the vicinity” of the measurement of interest. In the simplest case, Instrument 1 observes features of the “process of interest” *X*, as well as features of a single additional unrelated process (say *Y*); while Instrument 2 observes possibly the same or even different features of the same “process of interest” X, as well as features of an additional unrelated process, say *Z*, different from *Y*. This setup, involving measurements from two different sensors, is introduced in Fig. 1, and discussed in detail later in the manuscript. The paradigm is directly motivated from the important relevant work of Lederman and Talmon (16, 17), who used two cameras to observe three “dancing” robots (see Fig. S1); one camera observed Yoda (Y) and the Bulldog (X), while another camera observed the Bunny (Z) and a *different view* of the Bulldog (a different observation of *X*). This paradigm has the additional convenience that the images of each robot in each camera do not overlap and therefore “do not interact”: the “measurement channels” (the pixels of each camera) are what we will call “clean pixels”—they pertain to either the “common process” (the Bulldog) or to the particular camera’s “extraneous processes” (Yoda or the Bunny). The main result in Refs. (17, 18) was the development of an algorithm (the “Alternating-Diffusion” algorithm) that jointly processes the data from both sensor streams, and discovers a data-driven parameterization *of the common features across the sensors* (the measurements of the Bulldog). Alternating diffusion has also been applied to real-world data, such as electroencephalogram (EEG) and respiratory signals, demonstrating its usefulness in various higher dimensional (and noisy) biomedical applications, including sleep stage identification (16, 18, 19). Here we will use their computational technology as the basis for learning functions relating measurements of one camera to measurements of the other camera. That is, we will construct—when possible—observers of features measured by one sensor from features measured by the other sensor.

We will also briefly introduce and demonstrate another, more recently developed, algorithm for the extraction of the so-called Jointly Smooth Functions (JSF, (20)) as an alternative approach to the parameterization of the common features across two sensors (and therefore, as an alternative basis for learning cross-sensor observer functions). Other possible approaches to construct representations for common (and uncommon) coordinates between datasets are currently being explored. Coifman, Mashall, and Steinerberger (21) propose a framework to identify such coordinates across graphs, while Shnitzer et al. (22) propose antisymmetric operator approximation to encode commonalities and differences. The latter has recently been extended by incorporating the Riemannian geometry of symmetric positive definite (SPD) matrices (23, 24). In our work, we go exploit the results that our two approaches (as well as these latter ones) can extract, to learn functional relations (observers) across different observations of the same dynamical system.

We apply our two computational approaches to three distinct sets of nonlinear ordinary differential equations (our systems *X*, *Y*, and *Z*), observed from two sets of sensors: sensor 1 observes time series of variables in systems *X* and *Y*, while sensor 2 observes time series of variables in systems *X* and *Z* (hence, the common variables in our example pertain to the states of system *X*). Section 2 describes the systems we consider: (*X*) an autonomous limit cycle (periodic (25)), (*Y*) a periodically forced oscillator system (resulting in quasiperiodic dynamics (26)), and (*Z*) the Lorenz system (27) constrained to its (chaotic) attractor. Even though periodicity is present in these systems, it is not necessary for the approach to be successful; it *is* important though that the data should contain several (similar, not necessarily exact) recurrences of each observation.

In Section 3 (also see Section 2 of the Supplementary Material), we show how the data-driven parameterization of common features across sensors “discover” which sets of individual sensor 2 channels can be written as functions of some subset of sensor 1 channels, and *vice versa*. We demonstrate this learning process using several commonly available alternative methods: k-nearest neighbors (KNN (28)), geometric harmonics (GH (29, 30)), and feed-forward neural networks (FFNN (31)).

Having demonstrated the base case, later sections discuss potential problems and extensions. Section 5 discusses the case where individual sensor channels *do not* belong to observations of a single system (what we called “clean pixels”), but rather constitute a combination of observations of multiple systems (what we call “dirty pixels”). Specifically, we apply random linear transformations to each set of sensor data, so that each individual sensor channel variable is a linear combination of all measured variables from that sensor’s two relevant systems. Even in this more challenging setting, our computational approach can extract that system *X* is commonly observed by both sensors. In this case, one sensor’s observations cannot predict any particular channel of the other sensor; the second sensor channels are “unidentifiable” from measurements of the first sensor. Instead, we can describe *a level set* of the second sensor’s full measurement space that is consistent with the particular observations of the first sensor. We discuss how to parameterize such level sets using a manifold learning variant called *Output-Informed* Diffusion Maps (32, 33).

In Section 4, we consider the case when the channel measurements from sensor 2 include “future” measurements of variables measured “now” by sensor 1. This allows us to learn approximate evolution equations for the system that is common between the two sensors, establishing a certain type of causality between the two sets of measurements.

We conclude with further thoughts on the parameterization of the “uncommon variable” level sets, including the observation of common/uncommon features across scales, the possible use of new, conformal neural network architectures for this purpose, as well as good sampling techniques on these “uncommon” level sets.

## Illustrative examples

### Models of a periodic (X), a quasiperiodic (Y), and a chaotic (Z) response

To illustrate how manifold learning leads to finding common features across different sensor measurements and learning relations between them, we generated data from three independent nonlinear dynamical systems. For our common process *X*, we will use data from a surface reaction model studied by Takoudis et al. (25), which modifies the Langmuir–Hinshelwood mechanism by requiring two empty surface sites in the surface reaction step:


(1) $$\begin{array}{cc} & A+B⇌AS,\\ & B+S⇌BS,\\ & AS+BS+2S⇌4S+\text{ products}. \end{array}$$

After nondimensionalizing the rate equations, we obtain a system of two nonlinear differential equations in $\theta_{A}$ and $\theta_{B}$, the fractional surface coverages of the two reactants, and four parameters:


(2) $$\begin{array}{rl} & \frac{d\theta_{A}}{dt}=\alpha_{1}(1-\theta_{A}-\theta_{B})-\gamma_{1}\theta_{A}-\theta_{A}\theta_{B}(1-\theta_{A}-\theta_{B}{)}^{2},\\ & \frac{d\theta_{B}}{dt}=\alpha_{2}(1-\theta_{A}-\theta_{B})-\gamma_{2}\theta_{B}-\theta_{A}\theta_{B}(1-\theta_{A}-\theta_{B}{)}^{2}. \end{array}$$

This system exhibits sustained oscillations for certain parameter values; we will sample data from the limit cycle arising for $\gamma_{1}=0.001$, $\gamma_{2}=0.002$, $\alpha_{1}=0.016$, $\alpha_{2}=0.0278$.

For our first sensor-specific process *Y*, we will use data from a periodically forced version of the above oscillatory system: a forcing term with the nondimensional form


(3) $$\alpha_{2}=A_{0}+A\cos\;(\omega t)$$

is added, periodically perturbing the gas-phase pressure of B. For $\gamma_{1}=0.001$, $\gamma_{2}=0.002$, $\alpha_{1}=0.019$, $A_{0}=0.028$, A=0.002097, ω=0.01722, the long-term dynamics are quasiperiodic (26).

For our second sensor-specific process *Z*, we will use data generated on the attractor of the Lorenz system (27),


(4) $$\frac{dx}{dt}=\sigma (y-x),\;\frac{dy}{dt}=x(\rho -z)-y,\;\frac{dz}{dt}=xy-\beta z.$$

We use the parameter value set σ=10, $\beta =\frac{8}{3}$, ρ=28, which is known to result in chaotic dynamics.

We define our sensor setup so that the first sensor can only detect time series data of the variable $\theta_{A}^{X}$ from system *X*, and also of the variable with the same name, $\theta_{A}^{Y}$, from system *Y*. The second sensor can only detect time series of $\theta_{B}^{\left(X\right)}$ from system *X* and *y* from system *Z*. We include a time-delayed measurement for each channel, so that we can fully capture the dynamics of the common (periodic) system (in the spirit of Whitney (34, 35) and Takens (13, 36–38)), see Fig. 1:


(5) $$\begin{array}{rl} S^{\left(1\right)}(t) & =[\theta_{A}^{\left(X\right)}\left(t\right),\theta_{A}^{\left(Y\right)}\left(t\right),\theta_{A}^{\left(X\right)}\left(t-\Delta t\right),\theta_{A}^{\left(Y\right)}\left(t-\Delta t\right)],\\ S^{\left(2\right)}(t) & =[\theta_{B}^{\left(X\right)}\left(t\right),y\left(t\right),\theta_{B}^{\left(X\right)}\left(t-\Delta t\right),\;y\left(t-\Delta t\right)]. \end{array}$$

We take simultaneous measurements from each sensor at a sampling rate sufficiently faster than the frequency of our common system; due to their different frequencies/different natures of the responses, each system’s measurements cannot be long-term correlated with measurements of the other two systems.

The computational tools that will be used to process the data from these numerical experiments are discussed in Section 1 of the Supplementary Material. They include diffusion maps (and output-informed diffusion maps), alternating diffusion, jointly smooth function extraction, and local linear regression (LLR). The techniques for learning functions as a post-processing of the data analysis include k-nearest neighbors (KNN), geometric harmonics (GH), and “vanilla” (multilayer perceptron) feed-forward neural networks (FFNN). The corresponding algorithms are included in Section 2 of the Supplementary Material.

### Alternating-diffusion embedding

**Fig. 2.**
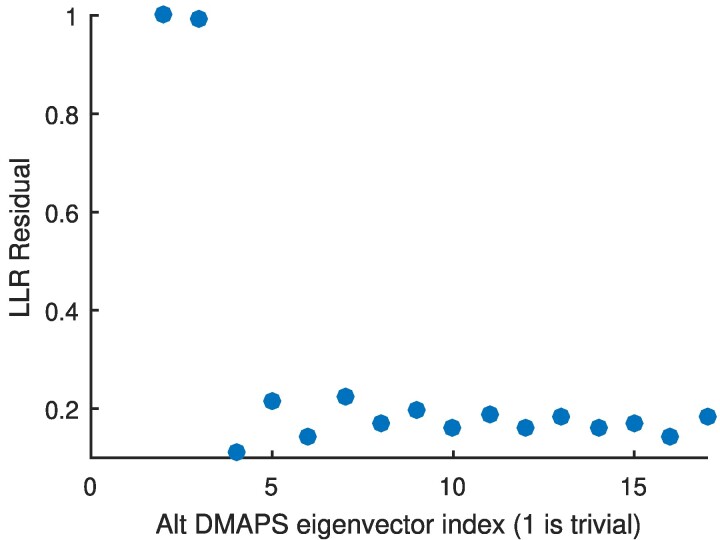
Results of running LLR on the set of successive alternating-diffusion eigenvectors $\phi_{i}$ (sorted by eigenvalue). $\phi_{1}$ is trivially constant, and $\phi_{2}$ has a normalized LLR residual of 1 by definition. $\phi_{2}$ is the only other top eigenvector with a high residual, indicating that it represents a unique direction and that the most parsimonious embedding of the common system is two-dimensional.

**Fig. 3.**
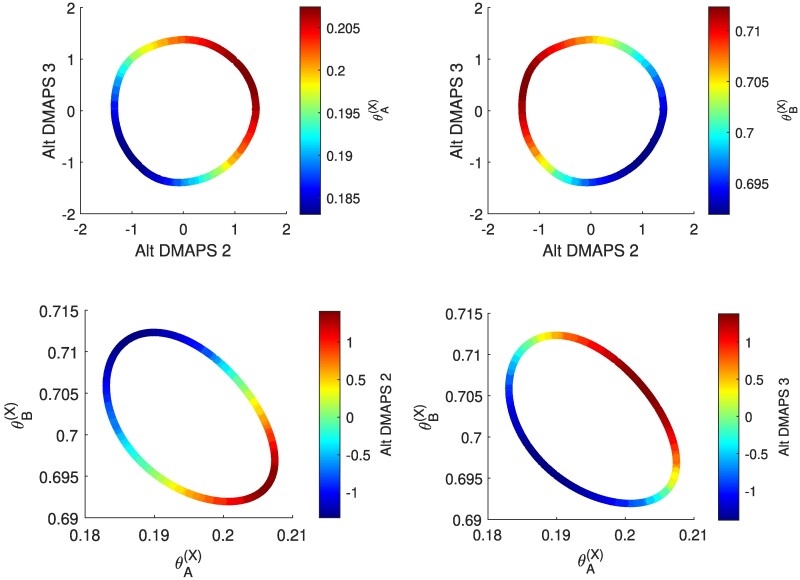
These plots confirm that the alternating-diffusion embedding is one-to-one/bi-Lipschitz with the coordinates of the common system X. (top) Plots of the alternating-diffusion embedding colored by $\theta_{A}^{\left(X\right)}$ (left) and $\theta_{B}^{\left(X\right)}$ (right). (bottom) Plots of $\theta_{B}^{\left(X\right)}$ vs. $\theta_{A}^{\left(X\right)}$, colored by alternating-diffusion eigenvectors 2 (left) and 3 (right).

We constructed our alternating-diffusion operator (17) as the product of two diffusion operators, each based on the Euclidean distances of the observations of sensor 1 and, separately, of sensor 2. We used LLR to analyze the true dimensionality of the recovered common coordinates and found that the first two nontrivial alternating-diffusion eigenvectors represented unique coordinates (see Fig. 2). In Fig. 3, we can visually confirm that the recovered common coordinates are one-to-one/bi-Lipschitz with the coordinates of the common system *X*.

**Fig. 4.**
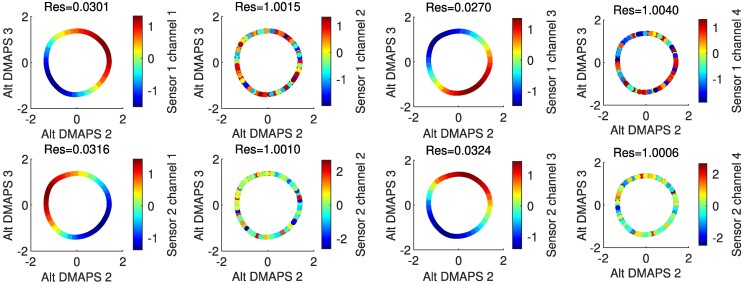
Plots of the alternating-diffusion embedding colored by each of the individual sensor channels, with the LLR residual above each plot. Channels 1–4 of sensor 1 (top row) are the measurements $\left[\theta_{A}^{\left(X\right)}(t),\theta_{A}^{\left(Y\right)}(t),\theta_{A}^{\left(X\right)}(t-\Delta t),\theta_{A}^{\left(Y\right)}(t-\Delta t)\right]$, while channels 1–4 of sensor 2 (bottom row) are the measurements $\left[\theta_{B}^{\left(X\right)}(t),y(t),\theta_{B}^{\left(X\right)}(t-\Delta t),\;y(t-\Delta t)\right]$. Coordinates that belong to the common system (sensor 1 channels 1 and 3, sensor 2 channels 1 and 3) have a low residual and appear visually smooth. Other coordinates have a high residual and appear noisy.

In general, alternating-diffusion does not require that each sensor channel (each camera pixel) involves observations of just one system (what we called “clean” channels or “clean” pixels above). Sensor channels that *combine* simultaneous measurements from the common system and one or more sensor-specific systems (what we call “dirty” channels or “dirty” pixels) cannot therefore be written as a function of our alternating-diffusion common coordinates (they are not *identifiable* from these common coordinates). However, in this current section, we will consider the case where (at least some) of our original sensor observations are “clean,” i.e. they only relate to our common system. We identify these “clean” channels using LLR (see Fig. 4). Later, in Section 5, we will also demonstrate how to extract *common* as well as *uncommon* coordinates if there are no “clean” observations available. Note also in Fig. 4 (col. two and four) that sensor-specific observations (pixels) *are not smooth functions of the common coordinates* (their Dirichlet energy appears visually extremely high). These measurements are clearly *not identifiable* from the common coordinates.

### Jointly smooth functions

We apply Jointly Smooth Functions (JSF) (20) to the same sensor data described above. In Fig. S5a, we visualize the first 10 JSF. As we can observe, only the first seven JSF are smooth (have low Dirichlet energy). Similarly to alternating diffusion, we can use LLR to select the two functions which give the most parsimonious embedding: they are the second and third JSF (nos. 1 and 2), whose relative shift is reminiscent of the shift between a sine and a cosine function. The common system coordinates are “nice” (low Dirichlet energy) functions of the chosen JSF (see Fig. S5b).

## Learning functions across sensors

Once we have found which measurements of one sensor stream “belong together” with which measurements of the second sensor stream, through their joint parameterization by common features, we can approximate, in a data-driven manner, the relation between them. In this section, we describe several approaches for achieving this function approximation: nearest neighbor search, geometric harmonics, and artificial neural networks. We demonstrate these methods on a dataset of samples including $\left(\theta_{A}^{X}(t\right)$, $\theta_{A}^{X}(t-\Delta t)$, $\theta_{B}^{X}(t))$. The first two coordinates are seen by sensor 1, and are one-to-one with the identified common coordinates. The last one is seen by sensor 2, and is a “clean” channel measurement: it should be possible to learn $\theta_{B}^{X}(t)$ as a function of the first two, i.e. $\left(\theta_{A}^{X}(t\right)$, $\theta_{A}^{X}(t-\Delta t)$. The dataset is split in training and testing subsets. For the training set, the values of $\theta_{A}^{X}(t)$, $\theta_{A}^{X}(t-\Delta t)$, $\theta_{B}^{X}(t)$ are known, while for the test set, we have only the values of $\theta_{A}^{X}(t)$, $\theta_{A}^{X}(t-\Delta t)$ and we will “predict” or “fill in” $\theta_{B}^{X}(t)$ values. For this section, we have used the first 50 sample data points for our function learning algorithms. The accuracy of each function learning algorithm is quantified based on the $L_{\infty }$ norm for $n_{\mathrm{samples}}=200$ values of $\theta_{B}(t)$,


(6) $$\varepsilon =\frac{\|\theta_{B}(t{)}_{\mathrm{true}}-\theta_{B}(t{)}_{\mathrm{predicted}}{\|}_{\infty }}{n_{\mathrm{samples}}}.$$

Fig. S6(a-c) shows the results when using KNN (a), GH (b), and FFNN (c) to map from two measurements of the common system *X* (measured by sensor 1) to a “clean” measurement of the common system *X* measured by sensor 2. All methods of approximation produce accurate extrapolation results on the limit cycle.

## Learning causality

Given the computational tools demonstrated in this work so far, we are now faced with an interesting possibility: if sensor 1 gives us measurements “now” and sensor 2 gives us measurements of the same quantities “in the future,” our common coordinates will allow us to learn quantities in the future *as a function of the same quantities now*—that is, help us learn a dynamical model of the common process. This brings us close to the idea (and the entire field) of data-driven causality.

A most basic premise to questions of causation is the principle that cause comes before the effect, but furthermore, a causal influence is one where the outcome is related to its cause. As simple as this concept may seem, it becomes nontrivial to develop a definition that is both robust but also testable in terms of data and observations. Two major schools of thought have arisen in modern parlance: the perspective of information flow, and the perspective of interventions. The information flow perspective includes the Nobel prize winning work on Granger-causality (39), and the recently highly popular transfer entropy (40) (TE), causation entropy (41–44) (CSE), Cross Correlation Method (CCM) (45), Kleeman–Liang formalism (46) and others, these being probabilistic in nature. In some sense, these all address the question of whether an outcome *x* is better forecast by considering an input variable *y* at a previous time, or not. If yes, then *y* is considered causal. However, the *intervention* concept, most notably developed in the “Do-calculus” of Pearl (47), is premised on a formalism of interventions and counterfactuals that are typically decided with data in terms of a specialized Bayesian analysis.

With the concept of common variables described in this paper, we are presented with the possibility of a different path to define causal relationships by asking the simple question as to whether observations of certain variables in the past are “common” with (contain sufficient common information to predict) observation of these variables in the future. By the data-driven methods developed here, we need only to prepare the data in the following manner: assume a stochastic process produces a sequence of vector valued data, $\{x(t_{i}){\}}_{i=s_{1}}^{s_{2}}$. Also, let $s_{1},\ldots ,s_{2}$ be a discrete index set, and $x(t)\;:\;R\to R^{d}$. In our wording, sensor 1 is shown multiple instances of past vector observations, $X=\{x(t_{i})\}$ and sensor 2 is shown multiple instances of the corresponding future observations $X^{\prime }=\{x(t_{i+1})\}$. Then the “common” coordinates connecting past and future may be understood as having a casual relationship. In these terms, clean observations of the common system by sensor 1 (now) are causally related to clean observations of the same common system variables by sensor 2 (the future): there exists a data-driven scheme that develops a nontrivial functional relationship from past observations to future outcomes. We are avoiding the phrase “correlate” because that has statistical connotations, usually assuming a linear relationship. Our common coordinate-based mapping from the present to the future is a deterministic, nonlinear one. Furthermore, while this machine learning/manifold-learning based approach is distinct from the Do-calculus there may exist a path to connect them: a bridge could be conceptually constructed if the data set itself includes some parametric interventions. Otherwise, it has aspects common to the Wiener–Granger causality concept of forecastability.

In our first setup, sensor 1 sees $\theta_{A}^{X}(t)$ and $\theta_{B}^{X}(t)$ from system *X*, and $\theta_{A}^{Y}(t)$ and $\theta_{B}^{Y}(t)$ from system *Y*. sensor 2 sees $\theta_{A}^{X}(t+\tau )$ and $\theta_{B}^{X}(t+\tau )$ from system *X*, and x(t+τ) and y(t+τ) from system *Z*, where here τ=200 time units, about 25% of the period of system *X*. By using time-shifted measurements, sensor 2 effectively sees “into the future” of system *X*, which will allow us to approximate the evolution equations for the system *X* variables.

**Fig. 5.**
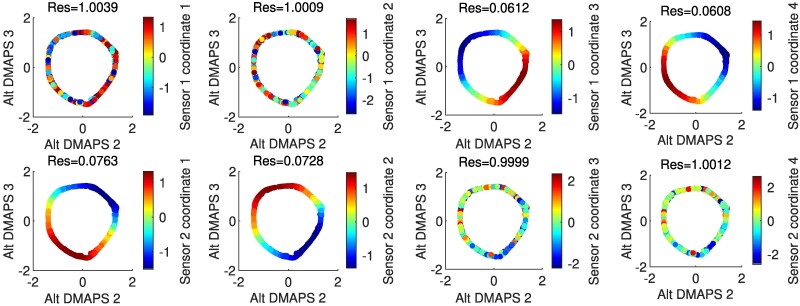
For the first setup, plots of the alternating-diffusion embedding colored by each of the individual sensor channels, with the LLR residual above each plot. Channels 1–4 of sensor 1 (top row) are the measurements $\left[\theta_{A}^{\left(X\right)}(t),\theta_{A}^{\left(Y\right)}(t),\theta_{A}^{\left(X\right)}(t-\Delta t),\theta_{A}^{\left(Y\right)}(t-\Delta t)\right]$, while channels 1–4 of sensor 2 (bottom row) are the measurements $\left[\theta_{B}^{\left(X\right)}(t),y(t),\theta_{B}^{\left(X\right)}(t-\Delta t),\;y(t-\Delta t)\right]$. Coordinates that belong to the common system (sensor 1 channels 3 and 4, sensor 2 channels 1 and 2) have a low residual and appear visually smooth. Other coordinates have a high residual and appear noisy.

**Fig. 6.**
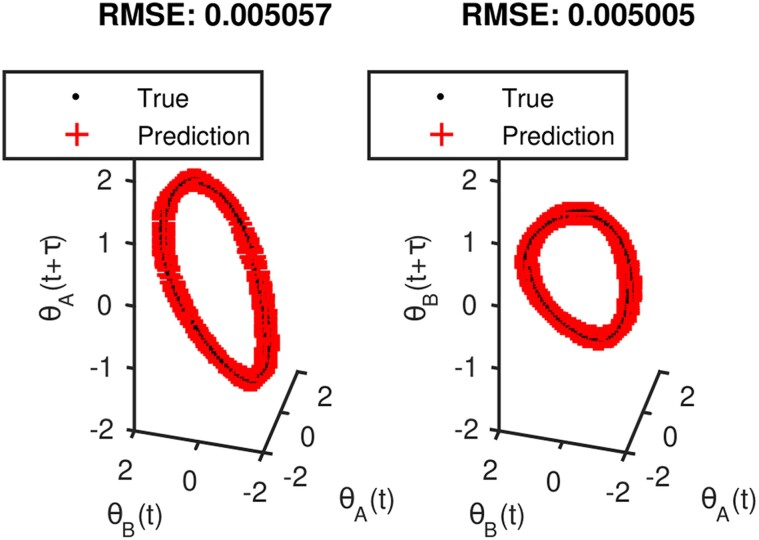
Learning causality with KNN. Here, we learn a map from $\left(\theta_{A}^{X}(t),\theta_{B}^{X}(t)\right)$ (the present) to $\left(\theta_{A}^{X}(t+\tau ),\theta_{B}^{X}(t+\tau )\right)$ (the future).

We use the LLR algorithm to determine that the alternating-diffusion embedding for this sensor setup is two dimensional. Visually, and the with LLR algorithm, we can determine which observables from each sensor are related to system *X*. In Fig. 5, the title shows the normalized residual value from the LLR algorithm. The variables which have residuals close to 0 are functions of the alternating-diffusion embedding, and thus can be assumed to only be related to system *X*. We can then learn functions from $\left[\theta_{A}(t),\theta_{B}(t)\right]$ to $\theta_{A}(t+\tau )$ and $\theta_{B}(t+\tau )$, effectively approximating the evolution equations. For example, the results from learning using a five-nearest neighbors regression are shown in Fig. 6. We can also apply Jointly Smooth Functions to the same sensor data described above. The results are presented in Section 3 of the Supplementary Material.

**Fig. 7.**
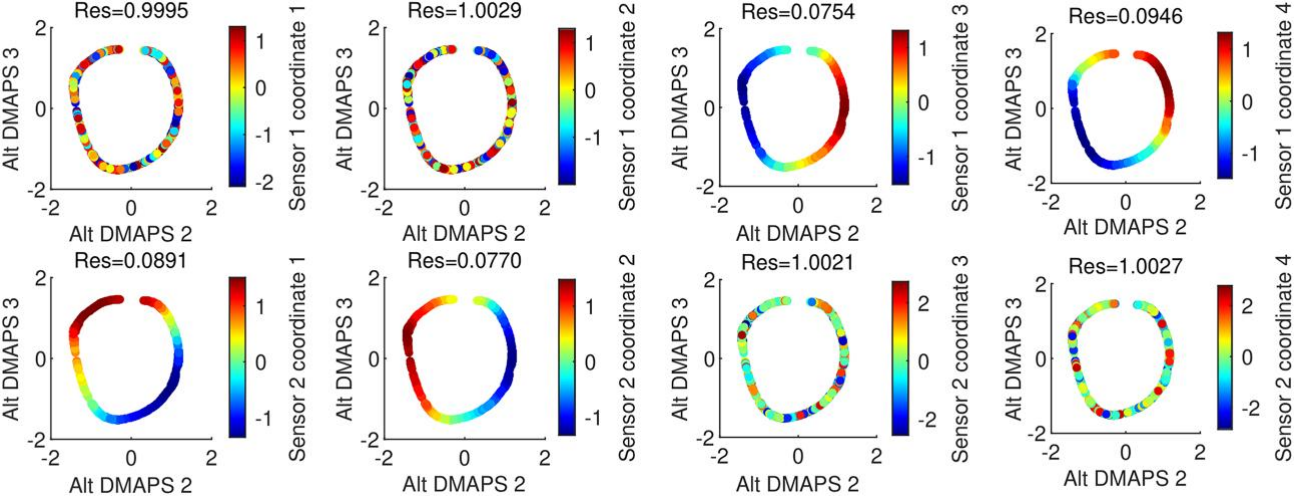
For the second setup, plots of the alternating-diffusion embedding colored by each of the individual sensor channels, with the LLR residual above each plot. Channels 1–4 of sensor 1 (top row) are the measurements $\left[\theta_{A}^{\left(X\right)}(t),\theta_{A}^{\left(Y\right)}(t),\theta_{A}^{\left(X\right)}(t-\Delta t),\theta_{A}^{\left(Y\right)}(t-\Delta t)\right]$, while channels 1–4 of sensor 2 (bottom row) are the measurements $\left[\theta_{B}^{\left(X\right)}(t),y(t),\theta_{B}^{\left(X\right)}(t-\Delta t),\;y(t-\Delta t)\right]$. Coordinates that belong to the common system (sensor 1 channels 3 and 4, sensor 2 channels 1 and 2) have a low residual and appear visually smooth. Other coordinates have a high residual and appear noisy.

**Fig. 8.**
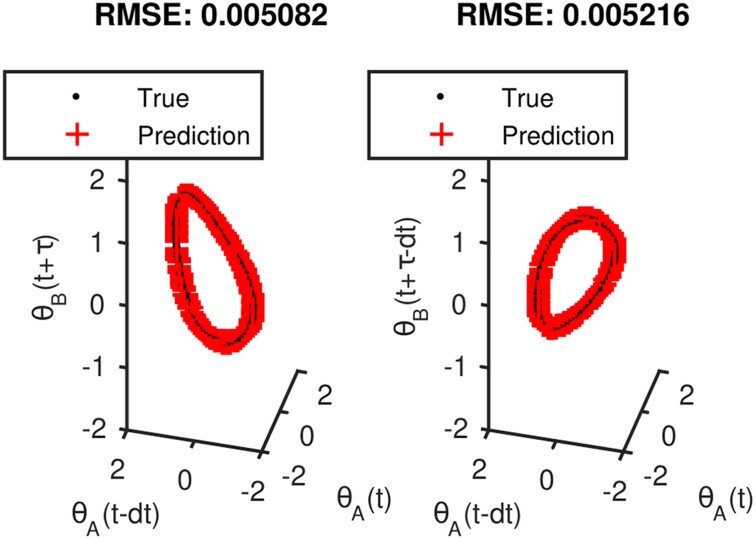
Functions from $\left[\theta_{A}^{X}(t),\theta_{A}^{X}(t-\Delta t)\right]$ to $\theta_{B}^{X}(t+\tau )$ and $\theta_{B}^{X}(t+\tau -\Delta t)$, constructed with a five-nearest neighbors regression.

For our second setup, sensor 1 sees $\theta_{A}^{X}(t)$ and $\theta_{A}^{X}(t-\Delta t)$ from system *X*, and $\theta_{A}^{Y}(t)$ and $\theta_{A}^{Y}(t-\Delta t)$ from system *Y*. Sensor 2 sees $\theta_{B}^{X}(t+\tau )$ and $\theta_{B}^{X}(t+\tau -\Delta t)$ from system *X* and y(t+τ) and y(t+τ−Δt) from system *Z*. Here, Δt=100 time units and τ=250 time units. Visually, and with the LLR algorithm, we show which observables from each sensor are related to each other (Fig. 7). We can learn functions from $\left[\theta_{A}^{X}(t),\theta_{A}^{X}(t-\Delta t)\right]$ to $\theta_{B}^{X}(t+\tau )$ and $\theta_{B}^{X}(t+\tau -\Delta t)$ with a five-nearest neighbors regression (Fig. 8). We can apply jointly smooth functions to the same sensor data described above and the results are shown in Section 3 of the Supplementary Material.

## Mixed sensor channels

What if our sensor measurement channels are “dirty,” meaning they involve combinations of measurements from the common and the sensor-specific observations? In this section, we apply the alternating-diffusion framework to sets of sensor data that are not directly separable into common and uncommon parts. All observations of each sensor are influenced by both the common and the sensor-specific system. Even in this setting, alternating-diffusion correctly uncovers a parameterization of the common system.

### Application to the oscillatory reaction example

Here the measurements of sensor 1 are linear combinations of all the “clean” sensor 1 channels—and the same thing holds for the measurements of sensor 2.

Beyond the mixing of the sensor 1 measurements, we use for sensor 2 measurements time-shifted by a fixed amount (approximately 25% of the period of system 2) *and* take linear combinations of them.

More explicitly, the sensor measurements are given by:


(7) $$\begin{array}{cc} & S^{\left(1\right)}=\begin{array}{cccc} \theta_{A}^{\left(X\right)}(t) & \theta_{B}^{\left(X\right)}(t) & \theta_{A}^{\left(Y\right)}(t) & \theta_{B}^{\left(Y\right)}(t) \end{array}\times \begin{array}{cccc} 0.3637 & -0.0173 & -0.3701 & 0.1013\\ -0.5068 & -0.4513 & 0.1470 & -0.2041\\ 0.0888 & -0.1818 & 0.3284 & 0.3344\\ 0.0407 & -0.3496 & -0.1545 & 0.3602 \end{array}\\ & S^{\left(2\right)}=\begin{array}{cccc} \theta_{A}^{\left(X\right)}(t+dt) & \theta_{B}^{\left(X\right)}(t+dt) & x^{\left(Z\right)}(t+dt) & y^{\left(Z\right)}(t+dt) \end{array}\times \begin{array}{cccc} -0.1394 & -0.0597 & 0.0828 & 0.3847\\ -0.3803 & -0.3576 & -0.3440 & -0.0628\\ -0.1010 & -0.5259 & -0.2147 & -0.3981\\ -0.3793 & -0.0568 & 0.3585 & -0.1544 \end{array}. \end{array}$$

**Fig. 9.**
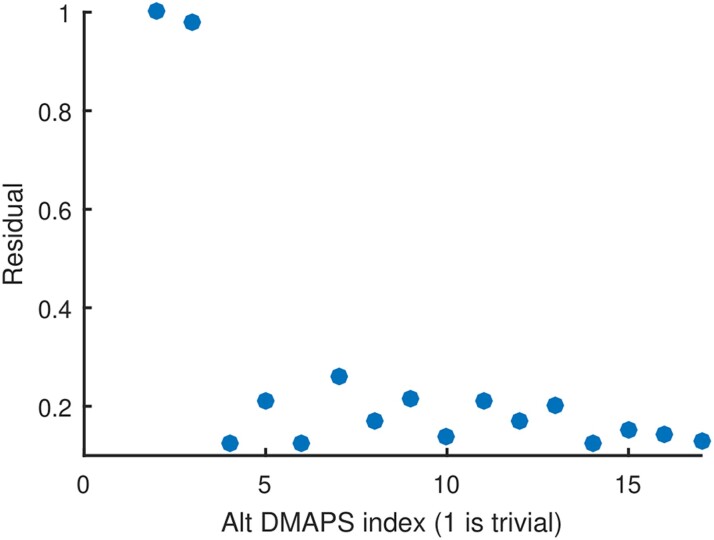
Results of running LLR on the set of successive alternating-diffusion eigenvectors $\phi_{i}$ (sorted by eigenvalue). $\phi_{1}$ is trivially constant, and $\phi_{2}$ has a normalized LLR residual of 1 by definition. $\phi_{2}$ is the only other top eigenvector with a high residual, indicating that it represents a unique direction and that the most parsimonious embedding is two dimensional.

**Fig. 10.**
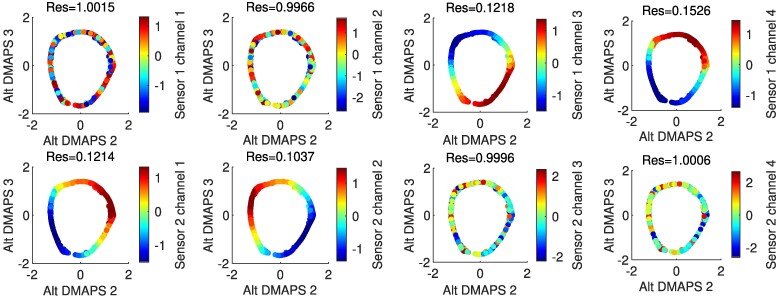
Plots of the alternating-diffusion embedding colored by each of the untransformed individual sensor channels, with the LLR residual above each plot. Channels 1–4 of sensor 1 (top row) are the measurements $\left[\theta_{A}^{\left(X\right)}(t),\theta_{A}^{\left(Y\right)}(t),\theta_{A}^{\left(X\right)}(t-\Delta t),\theta_{A}^{\left(Y\right)}(t-\Delta t)\right]$, while channels 1–4 of sensor 2 (bottom row) are the measurements $\left[\theta_{B}^{\left(X\right)}(t),y(t),\theta_{B}^{\left(X\right)}(t-\Delta t),\;y(t-\Delta t)\right]$. Coordinates that belong to the common system (sensor 1 channels 3 and 4, sensor 2 channels 1 and 2) have a low residual and appear visually smooth. Other coordinates have a high residual and appear noisy.

Here, Δt=200 time units, about 25% of the period of the common system. The resulting alternating-diffusion embedding is two dimensional (Fig. 9). Coloring the embedding by the *untransformed X* coordinates (Fig. 10) shows that we have indeed captured system *X*. We can apply jointly smooth functions to the same sensor data described above. The results are presented in Section 3 of the Supplementary Material.

## Output-informed diffusion maps

Even though the parameterization of the common system is discovered by either alternating-diffusion maps or jointly smooth functions, we cannot, in this case, learn a function from the common alternating-diffusion maps (AltDmaps) embedding to any of the individual original sensor channels. We can only say that points with the same AltDmaps embedding value *will lie on a particular level set* in the original sensor observation space. So, if we know enough information from sensor 1 to find where we are in the AltDmaps common embedding, we cannot tell what sensor 2 will simultaneously measure—but we can tell *what level set the measurements from sensor 2 will lie on*. Sensor 2 measurements are thus *structurally unidentifiable* from sensor 1 measurements in this case. For example, if *X* and *Y* are limit cycles with different (irrationally related) periods, and if sensor 1 measures at a particular phase of *X*, there will be many possible corresponding phases of *Y*—a one-parameter family of them—and they could be parameterized *by an embedding of the uncommon system*. To find this embedding, we can use a modification of diffusion maps, the so-called *output-informed diffusion maps* (32, 33), presented briefly here for clarity.

The goal of output-informed diffusion maps is to parameterize manifolds when variation along some directions on the manifold produces no response in some output measurement. In a typical scenario, the input manifold will be a sampling of the space of parameters for some dynamical system, and the output measurement will be the time series response of the system variables. If some parameter combinations are redundant (e.g. if only the ratio of two parameters influences the system response), the output manifold will have a lower-dimensionality than the input manifold. We would like to separate our parameterization of the input manifold so that the leading coordinates impact the system response, and they are followed by coordinates that do not. To accomplish this, we introduce a new kernel (proposed in a different context in the Thesis of S. Lafon (33), and also used in a similar identifiability context in (32)): let $f(y_{i})$ be the output response for input measurement $y_{i}$:


(8) $$w(y_{i},y_{j})=\exp\;\left(-\frac{\|f(y_{i})-f(y_{j}){\|}^{2}}{\epsilon^{2}}-\frac{\|y_{i}-y_{j}{\|}^{2}}{\epsilon }\right).$$

Since ϵ is typically less than one (or can be made so by scaling the original data), this kernel overemphasizes directions on the input manifold that actually result in changes in the output response.

In our case, we use the sensor data as the input manifold with the AltDmaps embedding as the “output,” which factors the standard Dmaps embedding of sensor 1 into *common* and *uncommon* eigenvectors. This also then gives us an embedding of the uncommon system (and an understanding of its dimensionality), as well as coordinates which parameterize the common level sets.

### Application to the oscillatory reaction example

**Fig. 11.**
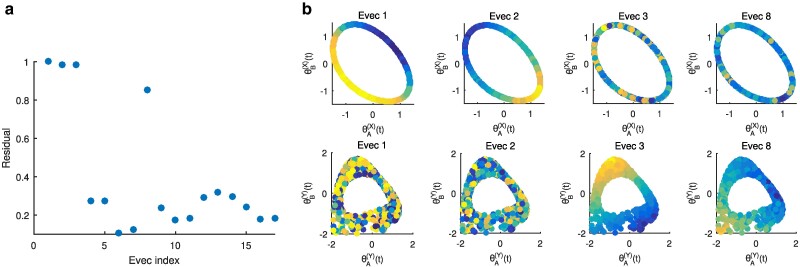
(a) Results of running LLR on the set of successive eigenvectors $\phi_{i}$ (sorted by eigenvalue) from output diffusion maps on sensor 1 data with the alternating-diffusion eigenvectors as the output. $\phi_{1}$ is trivially constant, and $\phi_{2}$ has a normalized LLR residual of 1 by definition. Eigenvectors 1, 2, 3, and 8 represent unique directions. (b) (Top row) Plots of the system *X* variables, colored by the output diffusion map eigenvectors 1, 2, 3, and 8. (Bottom row) Plots of the system *Y* variables, colored by the output diffusion map eigenvectors 1, 2, 3, and 8.

We can now use the alternating-diffusion embedding as the output response for output-informed diffusion maps, using sensor 1 as the input manifold. In the resulting embedding, eigenvectors 1 and 2 capture system *X*, *while eigenvectors 3 and 8 capture system *Y** (Fig. 11a and b).

We can also do the same thing using sensor 2 as the input manifold and the results are presented in Section 4 of the Supplementary Material.

In future work, we will use the parameterization of the *uncommon* manifold of each sensor to construct level sets of said sensor that are consistent with an observation set of the other sensor.

## Summary and outlook

We have demonstrated how we can find, in a data-driven way, common measurements between two (or, in principle, several) simultaneous measurement streams; our illustration was based on multiple observations (time series) from three nonlinear dynamical systems. This was accomplished through two alternative techniques: (a) alternating-diffusion Maps and (b) the construction of jointly smooth functions. Importantly, after the correlated measurements across the two sensor streams were detected, we could learn (in several data-driven ways) a quantitative approximation of their relation. We also showed how this approach can give us *a sense of causality*, helping uncover a data-driven dynamic evolution model for the common features. This suggest our first possible avenue of further research: it will be interesting to consider that the two (or more) sets of measurements come from different scale observations of multiscale systems (e.g. atomistic scale and continuum scale simulations of the same system). This should provide useful information regarding the appropriate level at which a useful closure should be attempted. We initially studied the “clean channel” case, where each measurement channel (pixel) comes either from the process of interest or (exclusively or!) the sensor-specific processes. We then proceeded to the “dirty channel” case, where each channel (pixel) contains a function of the both process of interest *and* sensor specific information. In this case, in principle, there is no identifiability across the observations: each set of measurements is consistent with an entire level set of measurements of the other. This provides a second possible direction of future research: given the probability distribution of the original data in their respective spaces, it should be possible—given a set of measurements from one of the observation processes—to construct not only the level set of consistent measurements of the other process, but also “the right” probability density *on* the corresponding consistent level set.

In this work, learning the transformation (in principle, a diffeomorphism) between corresponding measurements from the two (or more) observation processes was demonstrated—as proof of concept—using data science/ML techniques that are broadly available and used in the case of relatively few (say two, three, four) channels/dimensions. A true challenge lies in detecting the existence of, and constructing, these transformations in high dimensions, e.g. through solving functional equations or Hamilton–Jacobi–Bellman equations in high dimensions (48–54). The construction of modern computational techniques capable of this constitutes, by itself, an area of intense current research.

## Supplementary Material

pgae494_Supplementary_Data

## Data Availability

The data underlying this article and the codes used to perform the computations are available in a public repository from the authors at https://gitlab.com/eleni.koronaki/learningwhat2learn.git.
