# Supplementary material for: On learning what to learn: Heterogeneous observations of dynamics and establishing possibly causal relations among them
Source: PNAS Nexus. 2024 Dec 6;3(12):pgae494. doi: 10.1093/pnasnexus/pgae494 (PMC11630787; doi:10.1093/pnasnexus/pgae494)
Supplement: pgae494_Supplementary_Data [file pgae494_supplementary_data.pdf]

## Supplementary Information for

# On Learning what to Learn:

Heterogeneous observations of dynamics and establishing possibly causal relations among them

David W. Sroczynski, Felix Dietrich, Eleni D. Koronaki, Ronen Talmon
Ronald R. Coifman, Erik Bollt, Ioannis G. Kevrekidis
Corresponding Author: Ioannis G. Kevrekidis
Email: yannisk@jhu.edu

This PDF file includes:

- Supplementary text
- Figures S1 to S8
- SI References

# Supplementary Information Text

## 1 Computational Methods

This section briefly introduces the manifold learning technique “Diffusion Maps,” as well as a particular version of it, “Alternating Diffusion Maps” and the similar method of “Jointly smooth functions.” We also discuss a data-driven approach that helps decide whether a given data set can be described by a smooth input-output function: “Local Linear Regression”.

### 1.1 Manifold Learning: Diffusion Maps

The goal of manifold learning is to discover underlying nonlinear structure in high-dimensional data. Diffusion maps [2, 7] accomplishes this by constructing a discrete approximation of the Laplace-Beltrami operator on the data. When the data are sampled from a low-dimensional manifold, the discrete operator converges (at the appropriate limit of infinite sample points) to the continuous Laplace-Beltrami operator on the manifold. The discrete operator is constructed by defining a weighted graph on the sampled data, where the weight between points with indices $i$ and $j$ is given by

$$w_{i,j} = \exp\left(-\frac{d(\boldsymbol{y}_i, \boldsymbol{y}_j)^2}{\epsilon^2}\right), \tag{1}$$

where $d(.,.)$ represents a chosen distance metric, and $\epsilon$ represents a distance scale below which samples are considered similar. A weight of 1 indicates that two samples are identical, while a weight close to 0 indicates that two samples are very dissimilar. After some normalization, the eigenvectors $\phi$ of the weight matrix provide a new coordinate system to describe the data. Distances in this coordinate system are referred to as diffusion distances. Eigenvectors which do not contribute to this distance (due to low eigenvalues) can be truncated, and the reduced set of eigenvectors can serve as a proxy for the intrinsic manifold coordinates.

### 1.2 Alternating-Diffusion

The goal of alternating-diffusion[8] is to handle the situation where two multi-dimensional sensors measure information about the same underlying system, but observations from each sensor are distorted by sensor-specific, uncorrelated noise. More precisely, suppose that we have three independent systems which can be described by the high-dimensional variables $X$, $Y$, and $Z$. We do not have access to these variables, but rather to a set of simultaneous measurements from two high-dimensional sensors $S^{(1)} = g(X, Y)$ and $S^{(2)} = h(X, Z)$. We require that $g$ and $h$ be bi-Lipschitz functions. The alternating-diffusion algorithm defines two weight matrices, one based on the measurements from $S^{(1)}$ and one based on the measurements from $S^{(2)}$, and constructs the alternating-diffusion operator as the product of the two normalized weight matrices. It has been shown that the diffusion process defined by this operator is equivalent to one that would have been created from measurements of only the common variable X. More details can be found in Ref. [8]; we reproduce the procedure in Algorithm 1 and show a caricature example in Fig. S1.

### 1.3 Jointly Smooth Function Extraction

We now introduce a different, recently developed, kernel based data driven approach to extract common directions in data sets: that of *Jointly Smooth Functions* (JSFs) [3]. The JSF approach attempts to find functions of the individual sensor data sets that are *jointly smooth* across *all* the available data sets. We can then write all the common functions in terms of these JSFs, rather than describing the common parts of each data set as functions of each of the others.

Algorithm 2 constructs JSFs between $K$ data sets, arising from different observations of the same phenomenon, including sensor-specific (uncommon) noise. The key idea of the approximation procedure is to define function spaces on all $K$ data sets separately, through eigenfunctions of kernels. Then, we use

**Algorithm 1** Alternating diffusion map embedding from simultaneous sensor measurements.

**Input:** $2$ sets of $N$ simultaneous sensor measurements $\{\boldsymbol{S}_i^{(1)}, \boldsymbol{S}_i^{(2)}\}_{i=1}^{N}$ where $\boldsymbol{S}_i^{(k)} \in \mathbb{R}^{d_k}$.

1. Calculate two affinity matrices:

$$W_{i,j}^{(1)} = \exp\left(-\frac{\|\boldsymbol{S}_i^{(1)} - \boldsymbol{S}_j^{(1)}\|^2}{2\sigma_1^2}\right)$$

,

$$W_{i,j}^{(2)} = \exp\left(-\frac{\|\boldsymbol{S}_i^{(2)} - \boldsymbol{S}_j^{(2)}\|^2}{2\sigma_2^2}\right)$$

2. Compute diffusion operators

$$\tilde{W}_{i,j}^{(1)} = \frac{W_{i,j}^{(1)}}{\sum_{l=1}^{N} W_{l,j}^{(1)}}, \tilde{W}_{i,j}^{(2)} = \frac{W_{i,j}^{(2)}}{\sum_{l=1}^{N} W_{l,j}^{(2)}}$$

3. Compute the alternating diffusion operator $\tilde{W} = \tilde{W}^{(2)}\tilde{W}^{(1)}$

4. Compute a low-dimensional embedding based on $\tilde{W} = \tilde{W}^{(2)}\tilde{W}^{(1)}$

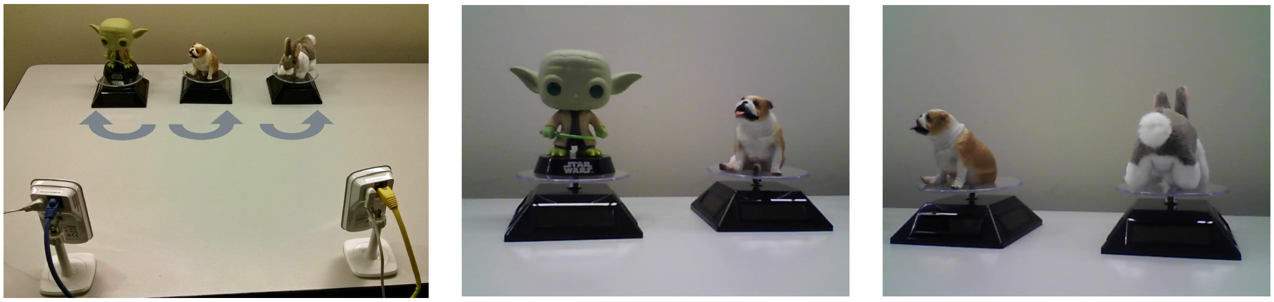

(a) (b)

Figure S1: The alternating diffusion algorithm is based upon data from two sensors. (a) A caricature setup where three figurines (Yoda, a bulldog, and a rabbit) are allowed to rotate independently and are photographed simultaneously by two cameras. (b) An example of simultaneous images from the two cameras. Although the bulldog is in the same intrinsic position in both images, the two cameras see different functions of its orientation. Alternating diffusion uncovers an embedding that is one-to-one with the intrinsic orientation.

singular value decomposition (SVD) to find the "common" functions across these spaces. For details on this approach, see the paper by Dietrich et al. [3]. Here, we have two data sets: $S^{(1)}$ and $S^{(2)}$. Therefore, we have to perform two eigendecompositions for two kernel matrices, and a subsequent SVD. The "common" functions between the two sensors $S^{(1)}$ and $S^{(2)}$ correspond to the common system (here, the limit cycle dynamics of system $Y$).

**Algorithm 2** Jointly Smooth Functions from $K$ sets of observations.

**Input:** $K$ sets $\{\boldsymbol{S}_i^{(1)}, \boldsymbol{S}_i^{(2)}, \ldots \boldsymbol{S}_i^{(K)}\}_{i=1}^N$ where $\boldsymbol{S}_i^{(k)} \in \mathbb{R}^{d_k}$.
**Output:** $M$ jointly smooth functions $\{\boldsymbol{f}_m \in \mathbb{R}^N\}_{m=1}^M$.

1. For each observation set $\{\boldsymbol{S}_i^{(k)}\}_{i=1}^N$ compute the kernel:

$$K_k(i,j) = \exp\left(-\frac{\left\|\boldsymbol{S}_i^{(k)} - \boldsymbol{S}_j^{(k)}\right\|^2}{2\sigma_k^2}\right)$$

2. Compute $\mathbf{W}_k \in \mathbb{R}^{N\times d}$, the first $d$ eigenvectors of $\mathbf{K}_k$.
3. Set $\mathbf{W} =: [\mathbf{W}_1, \mathbf{W}_2, \ldots, \mathbf{W}_K] \in \mathbb{R}^{N\times Kd}$
4. Compute the SVD decomposition: $\mathbf{W} = \mathbf{U}\Sigma\mathbf{V}^T$
5. Set $\boldsymbol{f}_m$ to be the $m^{\text{th}}$ column of $\mathbf{U}$.

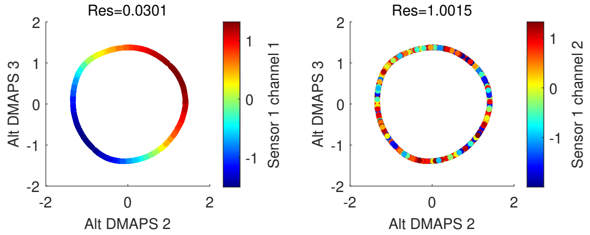


Figure S2: Example plots of an alternating diffusion embedding colored by two different sensor coordinates, with the LLR residual above each plot. On the left, the coloring is smooth, and LLR gives a low residual, indicating that this coordinate belongs to the common system. On the right, the coloring is erratic, and the residual is about 1, indicating that this coordinate is influenced by an independent, non-common system.

## 1.4 Local Linear Regression

When analyzing the eigenvectors of a diffusion operator, including the alternating-diffusion operator, simply discarding eigenvectors with eigenvalues lower than a defined threshold is not always sufficient to achieve the most parsimonious embedding. This is because higher harmonics of diffusion eigenfunctions are also eigenfunctions; e.g., $cos(x)$ and $cos(2x)$ are both eigenfunctions of the diffusion operator on a one-dimensional domain with no-flux boundary conditions. On multi-dimensional manifolds, the eigenvalues of these higher harmonics may happen to be higher than the eigenvalues corresponding to other unique coordinates.

To determine which eigenvectors of our discrete diffusion operator represent unique directions, we use local linear regression (LLR) as presented in Ref. [4]. We attempt to fit each successive eigenvector as a locally linear function of the previous eigenvectors, where locality is defined by a Gaussian kernel. For each sample point $i$, we determine our local fit coefficients by minimizing the sum of squared errors, but weighting the squared error at each training point based on how similar that point is to our test point:

$$\begin{gathered}\phi_k(i) \approx \alpha_k(i) + \boldsymbol{\beta}_k^T(i)\boldsymbol{\Phi}_{k-1}(i),\\ \hat{\alpha}_k(i), \hat{\boldsymbol{\beta}}_k(i) = \\ \underset{\alpha,\boldsymbol{\beta}}{\arg\min} \sum_{j\neq i} K(i,j)(\phi_k(j) - (\alpha + \boldsymbol{\beta}\boldsymbol{\Phi}_{k-1}(j)))^2.\end{gathered} \tag{2}$$

Eigenvectors with a low fit error are considered to represent higher harmonics of already known eigenvec-

tors, and can be discarded, while eigenvectors with a high fit error represent new unique directions. We will also use this method to determine which of our original measurements can be fit as functions of our intrinsic manifold coordinates. In the alternating diffusion case, this means that those coordinates "belong" to the common system. We show an example of coordinates belonging to the common system, as well as coordinates not belonging to it in Fig. S2.

# 2 Data-driven approximation of functions

We now describe the approaches we used to learn functions between our identified, common coordinates and the original measurements.

## 2.1 KNN

Typical regression methods are based on some *a priori* assumptions on the topology of data, as well as, say, the degree of polynomials in curve fitting. A collection of methods, known as "non-parametric regression" methods exist, for which knowledge about the shape of data is not necessary. The k-nearest neighbors (KNN [5]) method is a non-parametric technique, where the unknown label of a data point is estimated based on the labels of the $k$ nearest labeled points. In this section we first build a KDtree on the training data (with known labels). KDtree is a well-established algorithm for finding distances in high-dimensional data, where only similarity between close points are needed to be considered.

The location of a query (testing) point (with unknown label) is then identified in the constructed tree in $\theta_A(t)$ and $\theta_A(t-\Delta t)$ space (see Fig. S3(a)). The $k$ neighbor values of training $\theta_B(t)$ are then used to estimate the label of testing point. Here we used $k = 5$ neighbours. In Fig. S3(b) we show the true and prediction values for $\theta_B(t)$. The error is $\varepsilon_{kNN} = 9.6 \times 10^{-2}$.

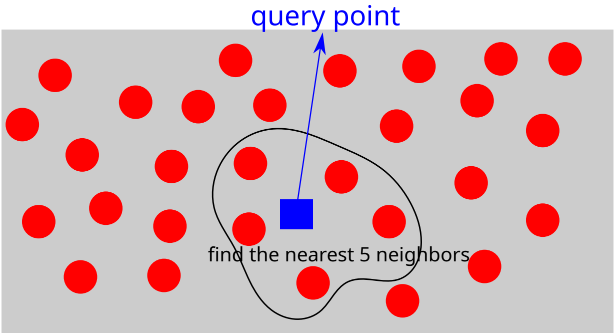


Figure S3: A schematic of the regression procedure with k-nearest neighbors. For any query point (blue square) in the test dataset, the five nearest neighbors (red circles in the loop) in the training set are identified. The label of the query point is then the weighted interpolation between the labels of neighbors.

## 2.2 Geometric Harmonics

Consider the case where we try to approximate a function $f(x)$ by another function $g_N(x)$, so that

$$f(x) \approx g_N(x) = \Sigma_{i=1}^{N} a_i \psi_i(x). \tag{3}$$

Here, $g_N$ consists of a sum over $N$ orthogonal functions $\psi_i(x)$ weighted by coefficients $a_i$. In one dimension, well-known orthonormal basis functions $\psi_i$ are sine and cosine functions, which arise in the context of Fourier series.

In previous sections, we presented diffusion maps (DMAPs) as a kernel learning method to find the intrinsic geometry of sets. Consider a (training) set $\Gamma$ subsampled from a larger space $\bar{\Gamma}$ with finite measure $\mu(\Gamma) < \infty$. The function $f : \Gamma \to \mathbb{R}$ is known and we are interested in approximating its value for some

$x \in \bar{\Gamma}$, $x \notin \Gamma$. This task is known as function extension/out-of-sample extension. With no *a priori* assumption on the geometry of $\Gamma$, one can choose many classes of functions for $\psi$. However, DMAPs can be used to set constraints on the feasibility of this extension based on the intrinsic geometry of the dataset. As the intrinsic geometry of $\Gamma$ is represented by DMAPs, the Nyström method will allow us to extend $f$ outside the set $\Gamma$ using a special set of functions known as Geometric Harmonics [7, 2]. These are based on eigenfunctions of kernels $k(x, y)$ in $\Gamma$, i.e., $\int_\Gamma k(x, y)\phi_j(y)d\mu(y) = \lambda_j\phi_j$. The basis $\phi_j$ is exactly the set of DMAP eigenfunctions, and can be extended to the Geometric Harmonics $\psi_j$ defined on $\bar{\Gamma}$ using the Nyström extension:

$$\forall\lambda_j > 0, x \in \bar{\Gamma}, \psi_j(x) := \frac{1}{\lambda_j}\int_\Gamma k(x, y)\phi_j(y)d\mu(y). \tag{4}$$

For $x \in \Gamma$ we have $\psi_j = \phi_j$, therefore $\psi_j$ are extensions of the basis functions $\phi_j$ from $\Gamma$ to $\bar{\Gamma}$. It can be shown that $\psi_j$ are orthonormal both in $\Gamma$ and $\bar{\Gamma}$ [2], and hence can be used to efficiently approximate functions away from the given data $\Gamma$. In this manuscript, $\Gamma$ is our training dataset on which we approximate five eigenfunctions $\phi_j$. Values of $\theta_B(t)$ on the test set in $\bar{\Gamma}$ are then approximated using Geometric Harmonics [1]. The error of the Geometric Harmonics regression based on (5) is estimated at $\varepsilon = 1.3 \times 10^{-2}$.

$$\varepsilon = \frac{||\theta_B(t)_{true} - \theta_B(t)_{predicted}||_\infty}{n_{samples}}. \tag{5}$$

### 2.3 Feed-Forward Neural Networks (FFNN)

In this section we use a multi-layered network of neurons to perform the regression task, i.e. learning $\theta_B(t)$ as a function of $(\theta_A(t), \theta_A(t - \Delta t))$. Our Feed-Forward Neural Network (FFNN) is shown schematically in Fig. S4(a). Consisting of two hidden layers with ten neurons each, it is first initialized by random weights and then weights are corrected during each epoch by error backpropagation. We implement the network in PyTorch [9], using the Adam optimizer for the correction of weights in each training epoch [6]. The “Randomized Leaky Rectified Liner Unit” (RReLU) serves as our activation function; it has the following form,

$$f_{act}(\alpha, x) = \begin{cases} \alpha x, & \text{for } x < 0 \\ x, & \text{for } x \geq 0 \end{cases} \tag{6}$$

In Fig. S4(b) we show the history of training in terms of Mean Squares (MSE) loss as a function of epoch.

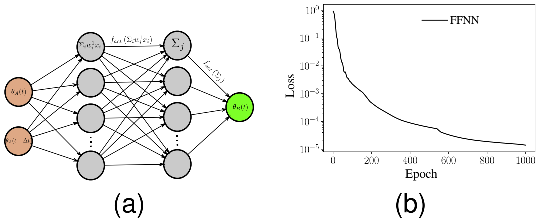


(a) (b)

Figure S4: (a) Schematic of Feed-Forward neural network with RReLU activation function. Inputs are training values of $\theta_A(t)$ and $\theta_A(t - \Delta t)$ and output is known values of $\theta_B(t)$. The network consists of two hidden layers with ten neurons in each layer. (b) The history of training in terms of MSE loss versus epoch.

After successful training we have used the network to find the values of $\theta_B(t)$ for the test dataset. The accuracy of prediction is shown in Fig. S4(c), while the error based on (5) is estimated at $\varepsilon = 3.8 \times 10^{-4}$.

# 3 Implementation of Jointly Smooth Functions

Here we present the results of the implementation of JSF to data from Setup 1 is presented in the Section titled “Learning Causality” of the main text. In Fig. S5a, we visualize the first 10 jointly smooth functions. Similarly to Alternating Diffusion Maps, we can use LLR to select the two functions which give the most parsimonious embedding (see Fig. S5b).

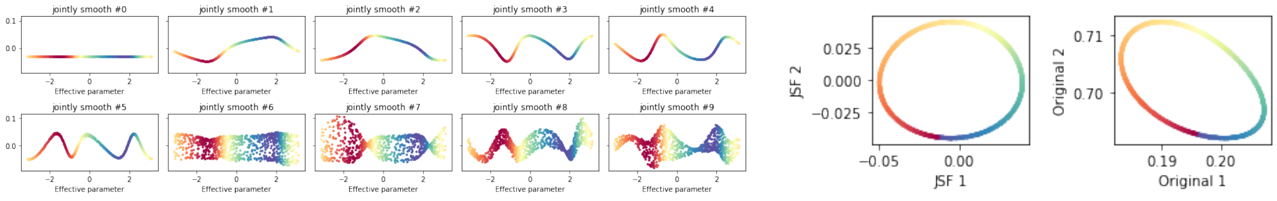


(a) (b)

Figure S5: (a) The first 10 extracted jointly smooth functions. (b)(Left)The embedding result for the two most parsimonious JSFs. *(Right)* The original system X data colored by one JSF.

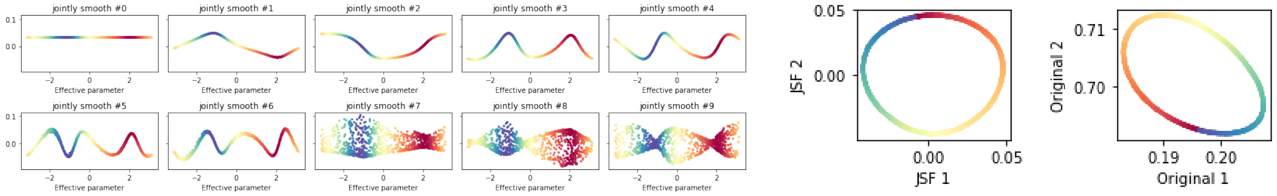


(a) (b)

Figure S6: (a) The first 10 extracted jointly smooth functions. (b)(Left)The embedding result for the two most parsimonious JSFs. *(Right)* The original system X data colored by one JSF.

JSF is then implemented to data from Setup 2 presented the in Section titled “Learning Causality” in the main text. In Fig. S6a, we visualize the first 10 jointly smooth functions. Similarly to Alternating Diffusion Maps, we can use LLR to select the two functions which give the most parsimonious embedding (see Fig. S6b).

The results from the implementation of JSF to data described in the Section titled “Output-informed Diffusion Maps” of the main text, is presented here. In Fig. S7a, we visualize the first 10 jointly smooth functions. Similarly to Alternating Diffusion Maps, we can use LLR to select the two functions which give the most parsimonious embedding (see Fig. S7b).

# 4 Output-informed DMAP results on Sensor 2

Similar to the implementation described in the Section titled “Output-informed Diffusion Maps” for Sensor 1, we present the results for Sensor 2 data. In the resulting embedding, eigenvectors 1 and 2 capture system X, while eigenvectors 3 and 15 capture system Y (Figs. S8a and S8b).

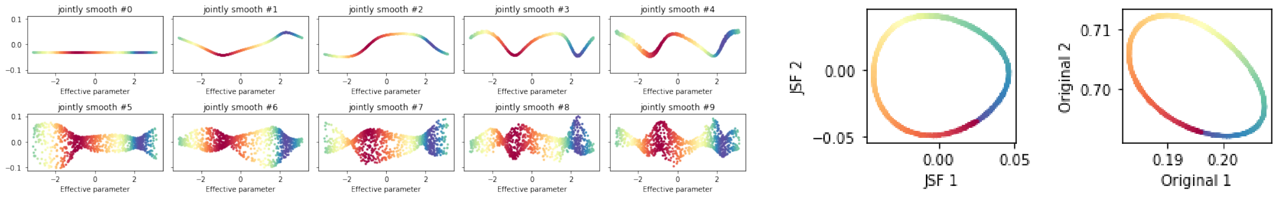


(a) (b)

Figure S7: (a) The first 10 extracted jointly smooth functions. (b)(Left)The embedding result for the two most parsimonious JSFs. *(Right)* The original system X data colored by one JSF.

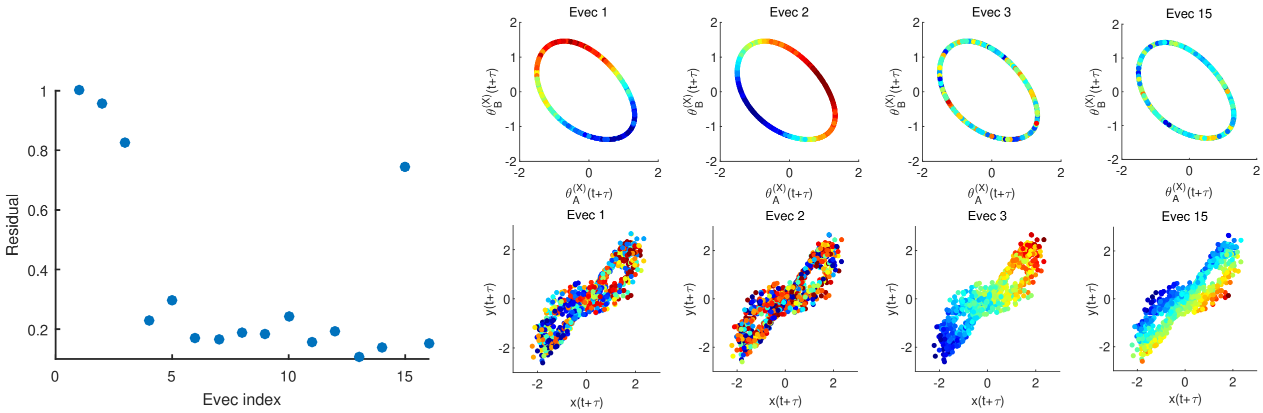


(a) (b)

Figure S8: (a) Results of running LLR on the set of successive eigenvectors $\phi_i$ (sorted by eigenvalue) from output diffusion maps on Sensor 2 data with the alternating-diffusion eigenvectors as the output. $\phi_1$ is trivially constant, and $\phi_2$ has a normalized LLR residual of 1 by definition. Eigenvectors 1, 2, 3, and 15 represent unique directions. (b)(Top row) Plots of the system X variables, colored by the output diffusion map eigenvectors 1, 2, 3, and 15. (Bottom row) Plots of the system Z variables, colored by the output diffusion map eigenvectors 1, 2, 3, and 15.

# References

[1] J. M. Bello-Rivas. jmbr/diffusion-maps 0.0.1. *Zenodo*, May 2017.

[2] R. R. Coifman and S. Lafon. Geometric harmonics: A novel tool for multiscale out-of-sample extension of empirical functions. *Appl. Comput. Harmon. Anal.*, 21(1):31–52, 2006.

[3] Felix Dietrich, Or Yair, Rotem Mulayoff, Ronen Talmon, and Ioannis G. Kevrekidis. Spectral Discovery of Jointly Smooth Features for Multimodal Data. *SIAM Journal on Mathematics of Data Science*, 4(1):410–430, March 2022.

[4] Carmeline J Dsilva, Ronen Talmon, Ronald R Coifman, and Ioannis G Kevrekidis. Parsimonious representation of nonlinear dynamical systems through manifold learning: A chemotaxis case study. *Applied and Computational Harmonic Analysis*, 44(3):759–773, 2018.

[5] Evelyn Fix and Joseph L. Hodges. Discriminatory Analysis. Nonparametric Discrimination: Consistency Properties. Technical report, Randolph Field, Texas, 1951.

[6] Diederik P. Kingma and Jimmy Ba. Adam: A method for stochastic optimization. *CoRR*, abs/1412.6980, 2014.

[7] Stéphane S Lafon. *Diffusion maps and geometric harmonics*. Yale University, 2004.

[8] Roy R Lederman and Ronen Talmon. Learning the geometry of common latent variables using alternating-diffusion. *Applied and Computational Harmonic Analysis*, 44(3):509–536, 2018.

[9] Adam Paszke, Sam Gross, Soumith Chintala, Gregory Chanan, Edward Yang, Zachary DeVito, Zeming Lin, Alban Desmaison, Luca Antiga, and Adam Lerer. Automatic differentiation in PyTorch. In *NIPS Autodiff Workshop*, 2017.
